# Chicago Medical Society

**Published:** 1889-01

**Authors:** 


### CHICAGO MEDICAL SOCIETY.

*Stated Meeting, November 5, 1888.*

President J. H. Etheridge, M.D., in the Chair.

Dr. Edward B. Weston read a

### PLEA FOR THE USE OF ANÆSTHETICS IN OBSTETRIC PRACTICE.

I recently had a patient, who sometime before confinement, of her own accord, brought up the subject, and requested me not to give her chloroform. I briefly explained matters to her, but told her I should of course respect her wishes. Though if the matter were to be left to me I should give her the chloroform. When labor at length began, and the pains of the second stage grew harder and harder, she quietly said, "I think I *will* have a little chloroform." She had previously given birth to one child without an anæsthetic; but she now said were she ever to have another, nothing could induce her to do without chloroform. I speak of this case to show that prejudice is disappearing, and I believe that in another five years chloroform will be the rule as it now is the exception.

An anæsthetic should be given to prevent suffering, if for no other reason. We have all seen cases where the child was small and the maternal parts of ample room, where the patient to all appearances, and from her own statement, did not suffer much. But even in such cases chloroform would have added to the credit side of the account.

I once had a patient who had a small tumor to be removed from his forehead. Against my advice, he declined to take ether. He bore the operation without the least indication of pain; but when it was finished said, with a groan, nothing would induce him to again endure what he had suffered. So in labor, in cases where the patient exhibits real bravery, and appears not to suffer, the pain is so great that we should consider it our duty to, and just as much expect to give an anæsthetic as if we were to perform a surgical operation.

I do not advise that all pain should be done away with—that from the first pain until delivery is accomplished not a pang should be felt. During the first stage chloral and opium find their place. But even then, if other means do not relieve from the wear of pains, which to the patient seem to do no good, a little chloroform is a great boon. During the second stage we should expect to relieve the patient from the greater part of her suffering, carrying the anæsthesia to complete insensibility if necessary. Of course the great majority of patients *can* endure the pain, if they have to. But unless there are physical objections, or objections on the part of the patient, we should expect and consider it our duty to give chloroform simply to save suffering. No one questions the propriety of an anæsthetic when an obstetric operation is necessary.

In a great majority of cases labor will be shortened if chloroform is used. The patient will be calmed and her courage increased; useless voluntary muscular action, and spasm, will be arrested and the uterus contract more regularly and with greater effectiveness.

But the greatest benefit is to be derived when the child enters the outlet and begins to distend the perineum. Then, in most cases, anæsthesia should be carried to the full surgical degree. The patient being unconscious, we may give her all necessary attention without undue haste, caused by our anxiety to save her from her horrible agony.

The patient being perfectly passive, the head descends and retreats regularly until the perineum is sufficiently stretched, the child is born and the mother probably uninjured.

To recapitulate:

1. An anæsthetic should be given to save suffering and an unnecessary expenditure of nervous force.

2. To shorten labor.

3. To enable the patient to pass through the completion of the second stage with less or no injury to the perineum, thereby saving an operation for repair, and a rent through which contagion from without may enter.

Anæsthetics will thus give us less necessity for the after use of antiseptics.

Dr. J. C. Hoag read a paper entitled,

MODERN VIEWS OF THEORETICAL AND PRACTICAL OBSTETRICS.

Assuming then that child-bed fever, so-called, is an infection differing so far as we know in no essential particular from surgical infection, and assuming further that we have to deal, not with an auto- but with an hetero-infection, what are we able to assert of the present state of our knowledge concerning the origin of this disease?

Puerperal fever, so-called, is to be regarded as a generic term practically the equivalent of surgical fever. Further than this we can only say that it is non-specific in character and may be produced by the streptococci as well as the staphylococci. Regarding the presence of micro-organisms in the genital canal of healthy women the following general deductions have been made by Dr. G. Winter from the experiments of the various investigators: 1. Bacteria of all sorts are found to some extent in the Fallopian tubes of the cadaver, while in those which have recently been removed from the living subject the bacteria are extremely few. 2. Bacteria of many sorts have been found in the uterus. 3. In the cervix and vagina great numbers of organisms of various forms are found, but none of these are pathogenic.

Winter's own experiments do not wholly confirm the above findings. Having examined quite a large number of tubes and uteri freshly removed from living subjects, he found that the normal tubes and healthy uterine cavity contain no micro-organisms, while in the vicinity of the os internum he found them in one-half of the specimens examined. The result of his examinations of the cervical secretions was, that in every healthy woman, numerous micro-organisms of three or four varieties, including both bacilli and cocci, are found. The micro-organisms become much more numerous during pregnancy, especially the bacilli. The vagina always contains micro-organisms in great abundance. He also gives in a table a comparison of the twenty-seven forms of bacteria, which he has found in the genital canal. Inoculation experiments with the three forms of staphylococci and streptococci which he identified, gave negative results. In his opinion the practical results of experimentation point to the necessity of the strictest measures of vaginal disinfection, because he believes that the germs present, though of weakened virulence, are able to reacquire their potentiality under certain indeterminate conditions and thus give rise to self-infection, so-called.

There is small room for doubt that the chief danger of infection, incurred by the puerperal patient, lies in the direct conveyance of noxious germs to the wounded surfaces of the genital tract, which are always present even after the easiest labor. It becomes then a matter of the utmost importance to take heed that whenever it is necessary to introduce the finger or an instrument into the patient's vagina, the same be most scrupulously disinfected. As we have no ready means of testing the perfection of our efforts at disinfection, and as we know that our hands are particularly difficult to disinfect, it is evidently most highly advantageous to our patients to spare them all unnecessary manipulation.

It is my purpose now to offer what I believe to be the essential desiderata of a well managed case of normal labor.

The prophylaxis of childbed fever should begin during gestation. The patient should live as far as possible in the midst of good hygienic surroundings and should bathe frequently, especially toward the end of gestation. The lying-in chamber should be well ventilated. The bed should be clean and a plentiful supply of sheets and towels should be provided. The external genitalia should be washed with an antiseptic solution after labor has set in, and care should be taken that the bladder and rectum be emptied. The physician should make as much as possible of the external methods of examination to the partial or complete exclusion of internal manipulations. The physician should always be provided with disinfectants and a nail-brush sublimate by preference, for the disinfection of his hands and arms, and carbolic acid for such instruments as may be needed. Above all other considerations I place the adequate disinfection of the physician's hands, and I am convinced that there are physicians whose hands have never been properly disinfected because their possessors are ignorant of what constitutes adequate disinfection.

Aside from all considerations of the patient's ultimate welfare, the genital tract should as far as possible, be protected from unnecessary lacerations, abrasion and contusions, as tending, of course, to securing a relative immunity from infection, and I am firmly of the opinion that these desiderata are highly promoted: First, by endeavoring to maintain the integrity of the membranes as so ably advocated by Dr. Henry Byford; and second, by causing the patient to maintain the lateral decubitus during labor, at least if she be a primipara. Perineal laceration may sometimes be avoided by a skillful employment of the method which enables one to promote the expulsion of the head, and is practiced by introducing the index and middle fingers through the patulous anus until the chin or mouth of the child is reached, and then exerting pressure in the direction of the symphysis. Furthermore, the head should be held back until the perineum has been suitably distended. It should be controlled, in fact, until the occiput has advanced quite under the symphysis, at which time it is in position to escape from the soft parts with its smallest diameter. The head should not be born during a pain, but in the intervals of the pains, and when the supra-orbital eminences are visible the perineum should be rapidly swept back over the face of the child by the physician's right hand, the left meanwhile being occupied in drawing the head back over the symphysis.

It is better not to tie the cord until after it ceases to pulsate, when it should be secured in two places, and cut between them, not only because of the possibility of a plural pregnancy, but because a placenta full of blood is in a more favorable condition for expulsion than a collapsed one.

One should not be over-anxious or impatient with regard to the third stage of labor, but should be content to wait until the uterine contractions have had time to loosen the placental attachments when the muscular action of the uterus may be aided by Créde's method of expression, or even by concomitant, though gentle traction on the cord after separation has taken place. The placenta having been expelled, it should be rotated a number of times so as to twist the membranes into a cord, with a view to effecting their complete removal.

To secure the complete extrusion of the secundines is a most important consideration, comparable even in its bearing upon the remote consequences to the woman, with that of managing the delivery of the child, without unnecessary laceration of the soft parts, for I believe that endometritis may often be directly traced to their imperfect expulsion at the time of labor. It has often occurred to me that the placenta comes away more readily and more perfectly in the case of primipara, though I am not sure that this is a general observation. To my mind, however, a placenta with a smooth, maternal surface, and without an excessive development of connective tissue, augurs well for the patient's subsequent health, to say nothing of its promise of a normal lying-in period, and accordingly when I am not satisfied that the secundines have been completely discharged, I endeavor to remove the fragments, often with the curette first, because a simple instrument can be more efficiently disinfected than the fingers, and secondly, because I can more thoroughly accomplish my purpose with its aid, especially in cases of abortion, where the placenta is apt to be especially adherent.*

If relatively extensive lacerations of the cervix or perineum have occurred they should be closed at once by a suitable operation, which, if carefully performed, yields excellent results. I have secured the complete union of a moderately lacerated perineum without even douching the vagina subsequent to the insertion of the sutures.

If the patient has entered upon a normal puerperium, after a satisfactory termination of labor, if no reasonable pains have been spared to shield her from the dangers of infection, and if the uterus has been completely emptied of its contents, there remains little to be done save to keep the patient clean by allowing the discharges to be absorbed by clean dressings (which should be frequently changed) and by washing the external genitals with antiseptic solutions. Uterine, and vaginal douches alike, may be safely dispensed with, unless distinct indications should arise for their use. The patient should be allowed to rise from her usual position in bed to evacuate the bladder and bowels, in order to favor the expulsion of blood clots, etc. It should be our aim to manage a case of labor with the idea that it is a physiological, and not a pathological, phenomenon.

If, however, in spite of all well-directed efforts to promote a normal puerperium by shielding the patient from the dangers of infection, the patient gives evidence of the dreaded symptoms which are so well known, there is no time to be lost. Watchful expectancy no longer avails. Activity is imperatively demanded. Prompt curetting of the endometrium has yielded excellent results in the experience of many physicians, and, having tried it myself, I have been extremely well pleased with the results. Its applicability, too, is by no means limited to cases where large masses of placental tissue have been retained, but, on the contrary, the most satisfactory results have often been obtained in cases where the third stage of labor was quite normal. Before and after using the curette the vagina and uterine cavity should be thoroughly irrigated with a suitable solution.

A single application of this method is often productive of the most satisfactory results. I have seen numerous cases where the temperature, previously very high, fell to the normal point in a few hours after this treatment, without subsequently rising. It is not necessary to suppose that in all these cases true sepsis is prevented, for the symptoms are often referable to putrid intoxication, and it is no doubt these very cases which are most markedly and most permanently benefited by curetting. I prefer the Sims' curette for most cases, believing that with many of the other forms it is impossible to reach all parts of the endometrium.

It is a matter of common observation that uterine douching once begun requires to be often repeated. The insertion of the uterine nozzle, however, is by no means always an easy procedure, and it is often attended with considerable pain to the patient. On the other hand curetting is a simple procedure, one which requires very little in the way of preparation, while it often accomplishes more than any number of uterine irrigations, and does not need to be repeated if properly performed. I have curetted the uterus after abortion without other assistance than that offered by the patient herself.

---

*Hoag. Some Considerations Regarding Death of the Fœtus in Utero. Trans. Chicago Gyn. Society, March, 1888.

Ergot is theoretically, and I believe practically as well, an important agent in the prevention of septic and toxæmic absorption. Quinine has long been held in high repute in these cases, but its efficacy has been positively denied by writers of eminence.

It is often necessary to direct treatment to the reduction of temperature. This may be well accomplished by the cold water treatment, or by the application of ice to the abdomen, in which situation its action is also favorable to the limitation of absorption, and to the prevention of tympanites. Antipyrin may be employed with good effect, its depressing action being guarded against by the free use of stimulants. There are possible sources of danger in the use of antipyrin from the fact that its chemical reactions are very imperfectly known. Mr. William Dyche, a skillful chemist of this city, informs me that he has observed the formation of an aniline green from its reaction with spirits of nitrous ether. He also observed a similar reaction in a mixture containing bromide of ammonium and bromide of potassium, though this occurred only when the ingredients were dissolved separately.

I have not had much experience in the treatment of peritoneal inflammation by the free administration of salines, but am inclined to regard it as a useful innovation on the old routine of "locking up the bowels" with opium.

There is a subject upon which I believe too little interest has been awakened in the discussions of the obstetricians, and that is the morbidity of patients. Almost every physician can glance over his records and produce a long list of obstetrical patients which did not die, and a very short list of the ones that did. But it is much more important to consider what proportion of the patients passed through a normal puerperium. Most of our information on this subject comes to us from gynæcological sources. I have no statistics to present, and can only say that I have often been astonished at the frequency with which one can find pelvic exudates, and their remains if he look for them. I have often examined 20 or 30 gynæcological cases in a day, nearly half of which gave evidence of this form of disease in greater or less degree. If, as some have claimed, the lying-in patient's attendants are to be held responsible for most cases of septic infection in its worst form, upon whom is the onus to fall in this other, and vastly larger, class of cases! This may not be an easy question to answer, but at all events a boast that that one has practiced obstetrics for a term of years without losing a patient from puerperal fever, does not amount to very much when one considers the origin of pelvic exudates.

In this connection I have often thought of the rôle played by the midwives who control such a large proportion of obstetrical practice. There are midwives in this city who boast of having attended 7, 8, and even 9,000 cases of labor. Some of them are doubtless admirably qualified for their occupation, but we have all, no doubt, seen midwives, who enjoy a large practice, and yet are wholly without the necessary qualifications for attending the simplest case of normal labor without exposing the patient to very grave dangers.

I have no wish whatever to pass criticism upon our civic authorities, but I have found food for meditation in the information that a knowledge of the uses of antiseptic agents forms no part of the requirements exacted of the midwives in this state. I do not know whether the relations are different in other parts of our country, but it has occurred to me that it would be both eminently useful, and entirely practicable, to furnish each midwife with printed instructions regarding the ordinary measures of hygiene to be observed in the lying-in room. Certainly the "effete monarchies of the east" protect their women with far more zeal than has ever been displayed in the

United States of America. In Germany and Austria, for example, not only are the midwives carefully qualified for their occupation, and closely watched in their conduct, but their duties are minutely explained in a book of instructions, and the greatest possible stress is laid upon the proper use of antiseptic agents, as well as upon more general points of hygiene. Judging from expressions which one often hears on the part of medical practitioners, I am by no means sure that a perusal of such literature would not be productive of great good to a certain class of physicians as well.

Dr. J. S. Knox: I was very much interested and very much pleased with both papers. I fully endorse the first one; I am an absolute believer in anæsthetics in labor. I believe many a patient suffers from a long, tedious lying-in simply from a painful labor, and I believe in the proper use of anæsthetics to control the pain; it gives the woman confidence and is an advantage both to her and her child. It has been a rule of mine to administer the anæsthetic as the child begins to extend the vulva and fully distend the perineum; always when I fear the rupture of the perineum. For an anæsthetic I never use anything but chloroform. I am satisfied of the ill effects of ether upon the kidneys, and as most lying-in women have a tendency to nephritic congestion, I think ether should never be used in labor, unless the woman is so depressed that chloroform is contra-indicated.

In the second paper I would find one fault, that is in regard to its advocacy of the curette. I am positively opposed to the growing use of the curette at term. A gentleman who has recently returned from Europe told me he had seen several autopsies on women who had been curetted after labor and in two instances he found the uterus perforated. I recently read, with great surprise, a paper by an obstetrician of this city in which she stated that she had perforated the uterine wall in one case, the lady leaving her office and going home, without ill result, and therefore she thought the curette was not dangerous, even if it did perforate the uterine wall. I do not see how it is possible to curettte with success the uterine cavity at term, without risk of perforation; if you remember that the uterine wall is only three-eighths of an inch thick after parturition, you can see that the curette would easily go through it. Then again my objection to the curette is, that I do not believe it accomplishes good if the uterus is fairly septic. I endorse most heartily the doctor's remark in regard to intra-uterine and vaginal douching. In normal labor I see no necessity for them. If the physician is thoroughly clean, disinfects his hands and manages the labor properly, the woman ought to go through without fever or sepsis, or douching. I agree with the doctor, that, where the indication arises for the intra-uterine douche, it should be used immediately, but I question as to what is the indication. How can one tell positively that the sepsis arises in the uterus? I confined a patient ten or twelve days ago who had suffered from neuralgia for several days, and was exhausted from loss of sleep and pain. Her labor was painful and instrumental, and after labor, within twenty-four hours she had pale features, a temperature of 105° and a good deal of abdominal tenderness. One would say these were indications for the intra-uterine douche—chills, extremely high temperature, sweats, pinched features, and tender abdomen; yet I was so positive that I had managed her case aseptically, and that her condition was due to nervous shock, that she was simply given stimulants and opium and kept warm, and in thirty-six hours she was comfortable. I am sure that if I had gone to work and douched her I would probably have inoculated her. If I had a positive indication that the uterus was septic I should certainly use the douche, but until positive I think there is more danger in the use of the intra-uterine douche than in its non-use. Our President saw with me a case of inversion of the uterus due to a large fibroid of the fundus. I was first called in consultation to see the case. The inversion occurred with excessive hæmorrhage. I succeeded in restoring the inversion and checking the hæmorrhage but could not get back what was apparently a portion of the fundus. When Dr. Etheridge saw the case the capsule of the fibroid had ruptured and it was undergoing decomposition, and during the next week the uterine cavity was constantly putrid. Intra-uterine and vaginal douches were used and she made an excellent recovery. The doctor who managed the case told me that during confinement he had thoroughly disinfected his hands with corrosive sublimate, and during all subsequent examinations the hands were made thoroughly aseptic. The intra-uterine douche, when it was used, could not destroy the putridity of that cavity while the fibroid slowly sloughed away and was discharged. If such conditions can exist in the uterus and the woman make a recovery, I fail to see the need of the intra-uterine douche for the ordinary febrile disturbances, so common during the lying-in period.

Dr. Frank Cary: I was very much interested in the first paper, but I can hardly agree with the author as to the use of anæsthetics so early in labor as I understood from the paper he recommends using them. As regards the majority of practitioners not using anæsthetics in labor, I think that it is true, but I think some of the younger men of to-day give chloroform to an extreme. I recently saw a patient to whom I found a young gentleman in attendance, giving chloroform nearly to surgical anæsthesia, in the first stage. As the case was mine he turned it over to me; I stopped the anæsthetic and it was nearly fifteen hours before the patient was delivered. As to using chloroform in the second stage when the head is passing over the perineum, I freely endorse that mode of treatment. As to saving more, or as many patients without antiseptics, I most surely do not believe it, for I think that antiseptics in the practice of obstetrics is fully as important as in surgery or gynæcology. Most of these patients after confinement are in a condition to absorb septic matter by reason of solution of continuity in different portions of the tract, as well as the denuded portion in the uterus after the removal of the placenta. As to the use of the curette, I agree in a great measure with Dr. Hoag. I should criticise Dr. Knox's remarks on the use of the curette, after the manner of Dr. Fenger while uniting a radius. Being told that it had been united by several other physicians, he replied "it makes a great deal of difference who does it." A physician may scrape a hole through the uterus with a curette; I know a gynæcologist in this city who has done it several times; but I do not think it is necessary. If the placenta has not thoroughly come away and sepsis has developed, I see no reason why we should leave that decomposing mass. But if we have removed it and got down to living tissue, then we can rationally use an antiseptic in the uterus. I do not believe in ever douching a patient unless it is called for, and by saying that it is "called for" I mean when the temperature goes up to 104 or 105, with offensive lochia and reason for supposing that we have decomposition going on in the uterus; I should most surely then use an intra-uterine douche. Dr. Knox said that in a patient he had the other day the temperature went to 105, there was delivery and collapse, but that he did not use an intra-uterine douche; I should most surely have done so. Moreover he said that if he had done so he would have inoculated the patient; I see no reason why he should have, if he had taken the proper precautions. Within the past year I had a physician come into my office and tell me he had just come from giving an intra-uterine douche, and three minutes after he told me he had been to see a patient with erysipelas. He told me afterward that he came near losing his patient. I believe that patients are frequently made septic by the nurse. Within the past year, in hospital practice, I had a case where the temperature ran up to 105. The most careful antiseptic precautions had been taken, and I said it was my fault or that of the nurse —I found that the nurse had been taking care of a septic case at the same time. I required that the vulva should be covered with an antiseptic dressing and the nurse had been changing that gauze after attending the other case which was septic. This patient had a temperature of 105, with pelvic exudate, which, however, subsided without suppuration.

Dr. William E. Clarke: I have used both ether and chloroform in labor, and I am sure from my observation that their use does retard labor to such an extent as at times to endanger the life of the mother and child. I am very sure of this, and yet the pressure is so great, the desire of saving pain occasionally compels me to use them. If, however, the patient will consent to wait till I advise an anæsthetic, there will be many that do without it. I have known cases where labor seemed to be suspended by the use of an anæsthetic, but upon its withdrawal, the pains came on, successfully terminating the labor. I was called a short time ago, in a case of prolapsus of the cord, where the patient was under an anæsthetic. When I arrived the pains had ceased, and I believe the chloroform had arrested the labor.

I agree most heartily as to the importance of antiseptics. Some things have been said in the discussion that I cannot quite understand. Why should a practitioner be so particular in the personal use of antiseptics and yet think that he could leave his patient with the uterus loaded with septic, decomposing matter, and expect a perfect recovery? In the case mentioned, it must have been disposed of, in some way, and I believe if it had been removed by the curette and douche it would have very much increased the chances of life. I use the douche when it is called for. A man would be silly and criminal to use it, or any other powerful instrument, when not called for. I use it whenever I think there is septic matter within the uterus. The results, as far as my observation goes, have been successful. I should use the curette when I thought it necessary. In fact I used it before I saw one made by an instrument maker. I made use of a double canula with a watch-spring passed through it, making a loop. Of course in using such an instrument, now, with our knowledge of sepsis, we should be very careful that the tubes of the canula were perfectly aseptic. With the movable loop you can not only scrape out the uterus, but engage and remove fragments of membrane or other matter that may remain.

Dr. L. T. Potter: Concerning the use of chloroform to the so-called surgical degree, in the second stage of labor, it strikes me that it is not advisable. The objections raised, and perhaps sustained, by the majority of the physicians to-night, that it retards the labor, is not the only objection. The objection to the use of chloroform is the great danger of post-partum hæmorrhage. Under the relaxed conditions of the muscles of the mother it is not essential in order to mitigate pain to give it to the surgical degree.

In criticising the second paper, Dr. Knox has said that he is unalterably opposed to the curette, but in favor of the douche. I am unalterably opposed to the douche, but in favor of the curette. In the use of the douche there is great danger, if there is a septic condition of the uterus, of driving, by a *vis a fronte*, that sepsis up the Fallopian tubes and giving rise to abscesses and other grave consequences. There is a proper and an improper use of every instrument, but if the curette is used properly, and with great care, it is of marked service in abortions, where, perhaps, the uterine walls are not quite as thin as immediately after labor at full term.

Concerning the use of antipyrin in reducing the temperature, I have experienced some grave symptoms in its use; whether it has been due to an impurely manufactured article, it is impossible for me to say. But I recognize that antipyrin is the product of coal tar, and, like carbolic acid, capable of producing a deleterious effect upon the kidney. I have already, in two cases, from the use of five grains of antipyrin every two hours, produced the same condition inside of eighteen hours: blood in the urine, and actual suppression of the urine, which, upon the withdrawal of the antipyrin, has been relieved and the normal condition of the kidney resumed. Concerning the other statements in Dr. Hoag's valuable and scientific paper, I most heartily endorse all he has said.

Dr. James G. Kiernan: It is well known that there are many conditions of mental excitement following labor. In the greater part of those cases the exciting etiological element has been, no doubt, the pain and anxiety attending labor, and very probably to the previous condition of the patient.

It has been my fortune—and my experience has been that of a number of others—to have seen a form both of melancholia and mania which was not a puerperal insanity, following labor, and which was prevented by the use of anæsthetics in subsequent labors. It seems to me that under such conditions the use of anæsthetics would be justified.

With regard to ether, I have seen three cases in which there was no doubt that the insanity was directly precipitated by the use of ether.

Dr. Hoag correctly remarks that antipyrin in combination with spirits of nitrous ether, has produced a peculiar greenish product, but more recent experiments have shown that this decomposition of the antipyrin was due, not to the nitrous ether, nor to the potassium bromide, nor to ammonia, but to the fact that both contain acid. When nitrous ether, made according to the United States Pharmacopeia, is used there is no precipitate. It should be remembered, too, in regard to bromide of potassium and ammonia, that both contain sulphurous acid. At the same time it is true that with the increased demand for antipyrin the color manufacturers who originally introduced the article, are making it with great rapidity, which produces a great tendency to decomposition, and the experience that it has produced poisoning, similar to carbolic acid, is not unique. But it should be remembered that there is no antipyretic but must, at some time, under some circumstances, produce untoward effects. These are likely to be either cardiac depression, or a peculiar skin eruption. In point of fact these peculiar conditions have followed the use of every one of the new antipyretics. They have been observed with quinine and thallin, and are very likely to follow the use of every other antipyretic.

Dr. C. W. Earle: It seems that one of the questions is in regard to the time we should commence to give an anæsthetic in a case of labor, and I quite agree with Dr. Cary that it is almost bad practice to commence too early. To commence during the first stage and fill the patient full of chloroform, when it is impossible for him to know whether the labor is to be terminated in one hour or twenty-four, is all wrong. He not only places the woman in a possibly dangerous condition during this stage of labor, but is giving an agent which, to my judgment, predisposes to post-partum hæmorrhage. I am not sure but that if the child is immature and puny, its life also is endangered. My practice usually is to commence the anæsthetic in the latter part of the second stage, when the head presses well against the perineum.

There is one question in regard to the use of opium during the post-partum period I wish to raise, although it is foreign to either of the papers. Do we endanger the life of the baby by giving an opiate to relieve the after-pains of the mother? I will tell you why I ask the question. I delivered a child on the South Side, in a family where the arrival of a girl baby was hailed with the greatest possible delight. On my second visit the nurse, who is one of the most intelligent and well trained nurses that I have on my list, told me the after-pains of the mother were rather severe, for which I did what I have done a hundred times before, prescribed deodorized tincture of opium in fifteen to twenty drops every two to six hours, according to pain. I believe this is a perfectly safe dose. That evening fifteen drops were administered, but the lady continued to have pains, and in two hours twenty drops more were given. The next morning the baby was discovered to be in a collapse, and in the afternoon died. I did not see the child, as I was attending another case, but I understand that it was cyanotic and convulsed, and the general impression made by the attending physician, with which I believe the nurse coincided, was that the opium taken by the mother caused the death of the baby. I would like to know the experience of other members of the society in regard to the administration of opium for after-pains. I took pains to look up the literature of the subject and found that it is the practice of every one, so far as I know, and yet I have learned of one other case, in the practice of a physician, where the child died of convulsions, and the only cause that could be assigned was a few drops of some of the anodyne preparations of opium given to the mother for after-pains. If anodynes given to quiet after-pains even in a minority of cases are dangerous to the babies it is time that the medical profession was aware of the fact.

In regard to antiseptic obstetrics, it is well-known to every member of this society that I believe antiseptic obstetrics can and is saving a larger number of lives than can be saved in both the departments of medicine and surgery. The mortality for a great number of years in hospitals has been fifteen per cent. or one hundred and fifty in every thousand. This has been so reduced that in the Vienna Hospital, where they confine ten thousand women annually, they lose only .58 of one per cent. What the mortality has been and now is in private practice is not, and it appears to me, can never be known. Dr. Hoag tells us that if some of the membranes or a very small piece of the placenta was left in the uterus he would either curette or give an intra-uterine douche, or remove it with his fingers. Why is this necessary? If the patient is absolutely aseptic, the nurse aseptic and the doctor aseptic, and all the surroundings aseptic why not let the debris alone to come away itself perfectly sweet and clean? I delivered a case recently where part of the membranes were left and I did nothing: I believed the woman would do better to be kept perfectly clean and let alone. I ordered an antiseptic pad to the vulva, occasional douches, and boracic acid or iodoform to the diseased tissues at the outlet, and in five days everything came away perfectly clean. Now I believe this woman was much better off than she would have been had I introduced my finger through the os and detached the membranes, or had given her a douche, or had curetted her. I am in favor of douches when they are required and in some cases always, particularly after operations have been performed.

Where a single stitch is put in the perineum the woman, in my judgment, will be safer if we give douches. It has seemed to me that where I restored the perineum and douches have been withheld, that the woman has not done as well as when they are given and repeated every twenty-four hours, for four or five days until every possible chance of damming up of the lochial discharge has passed away.

I believe in curetting, but I think the person doing it should know exactly what he is about, and I do not believe every person should be provided with a curette and proceed to do the operation at the first rise of temperature, unless there are special and definite indications. Sometimes a rise in temperature takes place when it is not necessary to curette. If it is done, however, it should always be performed with proper instruments, in a scientific manner and with all antiseptic precautions. Curetting after abortion or after labor at full term is altogether a different procedure. After abortion some most remarkable results occur. The recovery in most cases is particularly rapid. In some of these cases where the secundines and pieces of placenta remain, the temperature high and the woman going through the different stages of sepsis, a single curetting properly done (always giving an intra-uterine douche before and after), and covering the lacerated and bruised parts with either boracic acid or iodoform, will remove the patient from a dangerous condition to one of safe convalescence. After labor at full term there are large sinuses which are gradually closed by the thrombi. These thrombi are gradually organized and by the shrinking of the sinuses are obliterated. Now it has appeared to me that curettment of such a uterus may open some of these sinuses, may cause some of these thrombi to enter the circulation, producing alarming symptoms. This, then, is why I think curetting the uterus after full term should be carefully considered before it is performed.

Dr. A. E. Hoadley: I am a great friend to the anæsthetic in labor. I never refuse to give it when there is an indication for it. I am always perfectly willing to give it in the second stage, and my choice is ether. I make a cone suitable for the purpose, and pour in a little ether, and hand it to the patient, and instruct my assistant to put in a little as long as the patient can hold the cone. She is perfectly satisfied; she is conscious of every pain; it does not seem to retard labor. I have never had any ill effects from ether, and they can always take ether if they want an anæsthetic. After they have had a little ether and are getting along toward the close of the second stage, if I think there is any indication for full anæsthesia, four or five rapid inhalations of fresh ether will put the patient into a surgical condition of anæsthesia, or sufficient for practical purposes. I do not give chloroform often, not one case in ten where I do ether. I am satisfied with ether. Chloroform I have to administer myself, and watch it all the time. I have given ether almost continually for six days to an obstetric patient, without any ill effects. But then I do not approve of giving it early, and only give it when it is needed.

In regard to antiseptic obstetrics, I can only add my testimony in favor of the most thorough antiseptic precautions in obstetric practice, and when there is an indication of decomposing secundines in the uterus, I do not hesitate to remove them with the douche and curette. I almost always make use of cotton swabs with the douche. As I am douching the uterus I can wipe the entire surface of the uterus with the cotton. I am not afraid of wiping a hole through it, and I am not afraid of scratching a hole through it with the curette. It is only necessary to loosen these decomposed plates of membrane and wash them out. The physician who puts a sharp curette into a uterus after full term I think does an unjustifiable operation. A blunt curette, and that handled lightly, would, in my experience, be quite sufficient to remove the secundines, and I have good results follow such operations.

I will confess to a practice which, perhaps, is my own, that is in regard to removing the placenta. I commenced early in my practice to remove the placenta immediately after delivery, and have continued doing so up to the present time. I always intend to remove the placenta immediately after separating the cord. I do not wait for a pain. I never had any difficulty in removing the placenta. It comes away promptly, and without trouble to the patient. I am perfectly satisfied with the procedure, and shall probably continue to use it, with all due antiseptic precautions, of course, at the time. I remove it by gentle traction on the cord, and by hooking down one side of the placenta with the finger. In those cases in which it has been necessary to remove the placenta fifteen minutes or half an hour after the termination of labor, I have had difficulty, but immediately on separating the cord I can remove the placenta without difficulty; it comes away very easily.

Dr. L. A. Harcourt: I have attended about two thousand cases of obstetrics, and I know that I have not used either chloroform or ether more than fifty times. I know, too, that the cases in which it was not used made as good a recovery as those in which it was used. I am in favor of using it when there is an indication for it, but I recognize the fact that labor is a physiological process, and under ordinary circumstances no anæsthetic is required. I use it under two or three conditions: *First*, if the os uteri is rigid, and the pains excruciating, then I think an anæsthetic is useful. *Second*, if the perineum is rigid toward the close of the second stage in labor, an anæsthetic will help to prevent its laceration. *Third*, in puerperal convulsions, I certainly should use it.

I think the curette should not be used for the purpose of removing the entire placenta, but it is justifiable to use it to remove the debris. One of the speakers this evening said that septic fever is always the result of ignorance, or negligence, on the part of the practitioner. It seems to me that statement is a little too sweeping, and that there are other sources from which septic poison may arise. Many of the poorer classes call in their mothers, or sisters, or aunts, or neighbors, during the lying-in period, and these people hesitate, and almost refuse, to put anything clean about the patient. I invariably instruct them that nothing must be put near the woman that has not been thoroughly boiled, and is not absolutely clean, and I explain to them that there is no use in my trying to use antiseptic precautions if they do not, or the nurse does not. The two or three cases of puerperal fever I have had in 20 years I am satisfied I could trace directly to the attendant.

In regard to the administration of opium for after-pains, I have used it until within the last six years wherever it was required, and very seldom has it been required since I began practicing Crêde's method of removing the placenta. My patients hardly ever have an after-pain, and I believe if that method is successfully practiced, after-pains will grow beautifully less, and there will seldom be occasion for the administration of an anodyne to relieve them. It seems to me that in the case in question, there might have been some other cause than the administration of the deodorized tincture of opium. I have been in the habit of giving grain $\frac{1}{8}$ of morphia with what would be the equivalent of about gr. 7 of bromide of potassium every three or four hours, as required, and have never found any unpleasant symptoms result from the practice. In some cases I have given a good deal more, and frequently where there was a great deal of pain, or metritis, I have used a hypodermic injection of grain $\frac{1}{3}$ of morphia, without bad results to mother or child. In most cases the secretion of milk is rather scanty until about the third day, and in case the child dies on the third day, it could hardly have been poisoned by opium.

Dr. H. M. Scudder: If a patient takes chloroform well I feel perfectly safe in giving it early; as soon as the os has begun to dilate freely: and my experience has been that if carefully administered it will not interfere materially with the progress of the labor. If we begin to give chloroform early in the first stage, as we sometimes have to do with nervous patients, and those threatened with puerperal convulsions, we must be ready to use "Barne's bags," and to apply forceps, as the chloroform may seriously delay the labor.

I am strongly in favor of giving it in the second stage of labor. There has not been much said in reference to the way that it is given. I suppose it is usually given on a handkerchief. That is the way I used to give it, but I think the use of an inhaler is much more satisfactory.

By the use of the "inhaler" during the course of each pain, but not in the intervals, I think I am able to give my patients more relief from pain than I can by administering the chloroform in the ordinary way on a handkerchief, or by the use of a cone. I feel confident that the inhaler enables me to control the pain with less chloroform than I had to use when I gave it on a handkerchief. Although I have given chloroform in a large number of labors, I never had a case of serious puerperal hæmorrhage which I thought was attributable to the chloroform.

In reference to the curette, I agree more with Dr. Knox than with those who are very liberal in the use of it. I think the distinction Dr. Earle drew is a good one. There is not much danger in the careful use of the curette in cases of abortion. You can use the curette freely in abortion where you could not use it to the same extent in a puerperal uterus after labor at term. I prefer, before using the curette, to be perfectly sure that the uterus is septic. I prefer, generally, to use the douche first, and if this does not have the desired effect, to use the curette.

Dr. J. Frank: I cannot agree with the author of the first paper, that giving chloroform hastens confinements. My experience is that it retards it. I can safely say chloroform given to the surgical stage or near it decidedly retards labor.

I would recommend chloroform to be given with a great deal of caution to the stage of tolerance. If it is intended that the narcotic be given to the surgical stage, I would advise the accoucheur to keep his finger in the vagina on the uterus, or the hand on the abdomen, if the head is engaged under the pubis, as the safest and quickest means of knowing how the uterus is acting.

It might be said that there is danger of sepsis if the finger is kept in the vagina; if everything about the patient and the attendant, and everything used is antiseptically cared for, there is no more danger of infection from keeping the finger in the vagina, than there would be from any major surgical operation.

I am very glad that exceptions have already been taken by a previous speaker to a statement made by a critic on the second paper of the evening, that if puerperal fever is developed after a normal physiological labor, it is the fault of the physician attending. This is very strong language, and places every physician who undertakes a case of labor, in a delicate position, not only with his patient and her friends, but with the law. This question has caused hot debate in medical circles all over the world, and the conclusions therefrom have been: "It is not the physician's fault, unless he knowingly carries it to his patient." Noeggerath (some years ago) has shown that a great many cases of puerperal fever developing after confinement was due to a latent gonorrhœa in the woman, and I am fully satisfied in my own mind that there are many such cases. I have seen myself, two cases where puerperal fever was caused by a latent gonorrhœa in the woman, transmitted to her by her husband; the wife of course did not know anything about it. I had previously treated both of them for gonorrhœa. Both women recovered after weeks of illness.

As far as antisepsis is concerned, I am a thorough believer in it, not so much in the different chemicals used as to thorough cleanliness, no matter what is employed.

Dr. H. P. Newman: It would seem, in the discussion here to-night, that the objections were largely to the indiscriminate or improper use of anæsthetics, the curette and intra-uterine douche. It goes without saying that either of these three measures are harmful when injudiciously used, and certainly when used without discretion or proper indication, but they have their place in obstetric procedures. It has been said, too, that child-birth is a physiological condition. True! but our women, many of them, are in anything but a physiological condition at the time of child-birth. Particularly is this the case in cities, among women who live more or less artificially, and it is in this class of cases that we are called to use anæsthetics in a manner which would not perhaps be indicated in more robust, healthy women from the rural districts. I refer to the condition of nervous irritability which frequently occurs even in the beginning of labor. It has probably been the experience of every one here practicing obstetrics, that in certain women of a high nervous organization, the onset of the pains causes an overpowering dread and nervous excitement, which, acting through the sympathetic system, directly upon the uterine nerves of cerebro-spinal origin, disturbs their rhythmical action, preventing dilatation of the cervix, which contracts down upon the presenting part and arrests the progress of labor. Here I think is an indication for partial anæsthesia, that is if we use chloroform simply to allay this nervous irritability, and to quiet the anxiety of the mother; the inhibitory action of the nervous system will thus be overcome, and labor will progress, usually, without further interference. The same may be accomplished, possibly, by the use of other sedatives, perhaps an opiate as is popular with many physicians. But chloroform is indicated here and has done me good service.

The authorities, I believe, are generally united on the use of anæsthetics in the second stage, particularly in the latter part of this stage, and certainly profound anæsthesia should not be pushed before that time.

We ought to judge of our obstetrical cases not simply from our mortality list, but from the condition in which the woman is found subsequent to delivery. There is a vast amount of mischief done which of course does not result fatally, and which is not tabulated or reported. We flatter ourselves that if the difficult, or complicated case goes through without the loss of life it counts so much in favor of the judicious use of instruments, anæsthetics, the curette, douche, etc. I think this is a mistake. Those who practice gynæcology will find a vast number of these cases presenting themselves at the end of a few weeks or possibly months, with indurations and exudations about the pelvic organs, and with other evidences of mischief that occurred during the birth of the child and subsequently. These things ought to be considered in the judicious management of an obstetrical case, and not simply the mortality rate. I am in favor of the curette and intra-uterine douche when indicated and properly used, but their indiscriminate and injudicious use is to be deplored.

Dr. E. B. Weston: I think it is evident from the discussion this evening that anæsthetics are not used very freely and that the teachings of this society will not lead to their abuse, as the author of the paper on "Modern Views of Obstetrics" does not even mention them. It is also evident from the interpretation my paper has received that the members think I am very partial to them, which is a fact; but as I stated in the paper, in the first stage of labor chloral and opium have their place. That is not usually the place for chloroform, but in some rare cases where the patient is very nervous, almost convulsed, as you occasionally see them, then a few whiffs of chloroform will quiet them and the chloroform can be removed and the patient pass on for hours without its further use. I stated that even in the second stage anæsthesia was not to be pushed to the surgical degree until the last, except in rare cases. I stated that expressly; but I do believe that for a time immediately before the birth of the child the patient may be kept unconscious with perfect safety, and the pains go on with regularity, and the labor not be delayed. I believe the use of an anæsthetic in the final stage does save a great many patients, and does make the after use of carbolic acid or the bichloride of less importance; that is, if you can save an extensive laceration, as yon certainly can in a great many cases, you prevent the occurrence of an opening which will give ingress to septic material, and in that way you avoid puerperal conditions for which you afterward have to use antiseptics so freely. I would in some cases use the first stage of labor, and afterwards freely up to complete anæsthesia, if need be. But it is the patient's condition and not the stage of labor which should decide when and how to use the anæsthetic.

Dr. J. C. Hoag: There are a few things I wish to say. In the first place, with regard to the way in which I have been misquoted. I was very particular to say that I did not regard every case of infection as attributable to the physician. I do not believe it for a moment. I was also quoted as saying that the physician, and not the attendants, infected the patient. I used the word attendants purposely so as to include the nurse, mothers, aunts, neighbors, etc. The statement has been made here and elsewhere that in nearly all cases of septic infection it is due to the septic matter on the physician's hands or instruments. I have never made this statement.

My experience with the douche began about six years ago, and I have had rather an extensive experience with it. When I was still an interne in the hospital I was required to give intra-uterine douches of large quantities of water every three hours day and night, for several days, so that two patients was all that I could attend to. I found that it is not by any means an easy matter to give an intra-uterine douche in all cases; sometimes there is great difficulty in inserting the nozzle of the syringe, and a great amount of pain, as well, to the patient. So that the pain is less in treatment with the curette than with the douche, because the curette does once for all, whereas the douche has to be frequently repeated. The gentlemen at first seemed to point out the dangers of the curette very vigorously. I wish to have a few words to say on this subject. What they seemed to have in mind was a large, flabby uterus, that had not begun to contract, and that had extremely thin walls. I particularly pointed out the applicability of the curette in cases of abortion. Dr. Potter was the first to take this up and point out that the uterine walls in abortion are thick. I remember a case where I curetted the uterus after abortion, and finding the next day that the organ was still large, I wondered whether I could have left in there any considerable amount of placental tissue or any part of the fœtus. It did not seem possible, although the curetting was done under rather difficult circumstances. So I made an examination and passed my finger clear up to the fundus and got the uterine walls between my two fingers, and was perfectly astonished to find how thick these walls were. It would have been as difficult to force the instrument through that uterine wall as through a person's cheek. Blunt curettes have been spoken of, but a blunt curette may be of a very small diameter, and in that way it would be possible to pass it through the uterine wall. The instruments I speak of are very broad, and their scraping surface is more than half an inch, and it would be very difficult to pass an instrument like that through the uterine wall. The curette seems to some too difficult to use. I have not found it so. I have seen it used in the hands of very expert operators, and in the hands of very inexperienced operators, and I have learned as much from one class as from the other. It should be used with care, but I have not had any feeling of uncertainty in using it. I use it carefully, and control the uterus by holding it with a double tenaculum.

Dr. Earle, I think, rather took me up with regard to bits of retained membranes, etc. I think if the patient is properly attended in labor you can leave tremendous pieces of placenta and membrane in the uterus, without injuring the patient, if she is in an aseptic condition. I may say that the retention of placental tissue may give rise to bad results in the end, as sometimes they are retained indefinitely and result in the formation of polypi, or fibroid tumors. I have been interested in finding out why women abort so frequently. We generally attribute it to traumatism, but I do not think that is the case. I think abortion is much more common from other causes, and particularly so from disease of the placenta itself. I think the placenta should be removed immediately at the termination of labor, or, at least, in a few hours, and not left there indefinitely. The danger I was getting at was the *remote* danger to the patient. I think Dr. Hoadley must have been exceptionally fortunate in his cases. He apparently has never run across an adherent placenta where there is a great proliferation of connective tissue, so that it is almost impossible to get it away entirely. I remember a case where I curetted a woman who had aborted at about the fifth month, and the placenta remained there twelve or fifteen hours. I curetted then, not so much to get it out immediately to prevent infection, as for the fear that hæmorrhage would be set up. Dr. Harcourt thought the curette should only be used to remove the *debris* of placental tissue; but if a woman aborts in the early weeks of pregnancy the *entire* placenta may be there, and I do not see any counter-indication to removing all of it. In the case where I curetted, fearing hæmorrhage, after fifteen hours, the patient had had pretty strong contraction pains, but there was no loosening of the placenta, and yet I had no difficulty in removing the whole placenta without introducing the hand into the uterus. The patient made a beautiful recovery. I did not care to go into the pathology of puerperal infection very extensively, but I do not think any of us would have difficulty in finding out where the inflammation was; if the patient's parametrium was filled with exudate I do not think any of us would be inclined to curette the endometrium with a view to prevent an infection which had already taken place.

